# Recent research progress in tetrodotoxin detection and quantitative analysis methods

**DOI:** 10.3389/fchem.2024.1447312

**Published:** 2024-08-14

**Authors:** Chao Lin, Qirong Li, Dong Liu, Qiang Feng, Hengzong Zhou, Bohe Shi, Xinxin Zhang, Yurui Hu, Xinmiao Jiang, Xiaoming Sun, Dongxu Wang

**Affiliations:** ^1^ School of Grain Science and Technology, Jilin Business and Technology College, Changchun, China; ^2^ Laboratory Animal Center, College of Animal Science, Jilin University, Changchun, China

**Keywords:** tetrodotoxin, detection methods, immunosensors, aptamer, LC-MS

## Abstract

Tetrodotoxin (TTX) is a highly potent and widely distributed ion-channel marine neurotoxin; it has no specific antidote and poses a great risk to human health. Therefore, detecting and quantifying TTX to effectively implement prevention strategies is important for food safety. The development of novel and highly sensitive, highly specific, rapid, and simple techniques for trace TTX detection has attracted widespread attention. This review summarizes the latest advances in the detection and quantitative analysis of TTX, covering detection methods based on biological and cellular sensors, immunoassays and immunosensors, aptamers, and liquid chromatography-mass spectrometry. It further discusses the advantages and applications of various detection technologies developed for TTX and focuses on the frontier areas and development directions of TTX detection, providing relevant information for further investigations.

## 1 Introduction

Tetrodotoxin (TTX)–a low-molecular-weight, crystalline, nonprotein organic compound with weakly basic properties and a colorless appearance–ranks among the most potent marine toxins studied to date (Katikou et al., 2022). It is a perhydroquinozolineamine molecule (aminoperhydroquinazolone), and its structure has been elucidated (Tsuda et al., 1964). Named after the family Tetraodontidae (including puffer fish), TTX was originally isolated from the puffer fish and has since been detected in other marine and terrestrial species (Lago et al., 2015). However, the biosynthetic process or biological origin of TTX remains unclear. Some studies have suggested that TTX mainly originates from bacteria belonging to the phylum Proteobacteria living in marine animals, including *Pseudomonas*, Pseudoalteromonas, and *Vibrio*, while others have found that at least 150 bacterial strains isolated from various organisms can produce TTX (Magarlamov et al., 2017). However, due to the limitations of analytical techniques, the number of bacterial species that produce TTX is still unclear. In addition, the origin of TTX is still under debate, and current mainstream research indicates that it may either be produced by symbiotic bacteria (endogenous pathway) or arise *via* exogenous accumulation through the diet (Biessy et al., 2019).

Voltage-gated sodium ion channels are pivotal in regulating neuronal excitability and maintaining the resting potential. They are indispensable for both the initiation and transmission of action potentials within neurons (Wang et al., 2017). The toxic mechanism of TTX involves binding to these channels and selectively blocking their action potential along nerves, skeletal muscle, and the myocardium, thereby reducing the membrane excitability of vital tissues, cardiomyocytes, skeletal muscle, and the central and peripheral nervous systems (Sorokin, 1973). The severity of symptoms caused by TTX depends on the dose, with patients typically experiencing toxic effects within 30 min to 6 h after ingestion. These symptoms range from headaches, sweating, and numbness to dysphagia, nausea, vomiting, abdominal pain, general discomfort, weakness, and a lack of coordination. In severe cases, individuals may also present with low blood pressure, arrhythmia, muscle paralysis, cranial nerve dysfunction, and potentially fatal respiratory and/or heart failure. TTX is highly toxic to all mammalian species, with the estimated minimum lethal dose in humans at approximately 10,000 MU, or ∼2 mg (Noguchi and Ebesu, 2001). Currently, no clinically proven antidote is available for the effective treatment of TTX poisoning (Narahashi, 2001). The clinical management of TTX poisoning in humans primarily involves symptomatic care, including supportive measures such as inducing vomiting, gastric lavage, providing respiratory support, and ensuring adequate hydration until TTX is eliminated from the body through urine (Sims and Ostman, 1986). In addition, antiserum and monoclonal antibodies targeting TTX have been developed and tested; however, further research is required to fully understand their clinical efficacy (Xu et al., 2005). Notably, due to the mechanism of action and pharmacological activity of TTX in the human body, clinical applications of TTX as an analgesic, anesthetic, and antitumor drug have been developed.

Most cases related to puffer fish poisoning mainly occur in Asian countries, such as Japan and China. However, as puffer fish and other TTX-carrying organisms continue to spread globally, facilitated by the expansion of international aquaculture trade, TTX poisoning is transcending its historical confinement to Asian countries and emerging in broader geographical regions, including the Pacific and the Mediterranean. Consequently, the incidence of TTX poisoning cases is anticipated to escalate further (Noguchi and Arakawa, 2008), the challenges of which include a high mortality rate and the absence of a specific antidote. Mitigating the risks associated with TTX poisoning requires taking essential precautions as the primary means of prevention (Saoudi et al., 2011). TTX has become one of the routine test indices significant in public health and import and export inspections and quarantines. Therefore, the development of effective analytical methods and rapid detection technology for TTX determinations will not only aid in conducting more in-depth research on the detoxification and application of TTX and its analogues but also ensure the safety of seafood and effectively prevent the occurrence of TTX poisoning. This review summarizes a variety of methods for detecting and quantifying TTX, including bio- and immunoassays; cell, electrochemical, and aptamer sensor assays; and instrumental assays.

## 2 Detection based on biological and cellular sensors

The mouse bioassay (MBA) serves as the official method for detecting TTX. While it boasts simplicity and instrument-free operation, its applicability for the routine monitoring of marine toxins is hindered by, for example, low sensitivity, inconsistent reproducibility, a lack of specificity, and ethical concerns (Reverte et al., 2015). Nevertheless, because this method can directly provide toxicological information, some current research efforts continue its use. Its implementation involves intraperitoneally injecting the extracted TTX diluent into male ddY mice weighing 18–20 g. The fatality rate is expressed in MU, where 1 MU is defined as the amount of toxin that kills mice in 30 min (Asakawa et al., 2019). A study assessed the tissue distribution following oral TTX in mice, revealing that the elimination cycle of a single oral TTX dose (75 μg/kg) was approximately 168 h. Continuous oral TTX administration demonstrated dose-dependent toxic effects on the liver and kidneys, offering valuable information for the management of TTX toxicity following low-dose ingestion (Zhong et al., 2023).

Cell-based biosensors, utilizing living cells as sensing elements alongside sensors or transducers, offer advantages such as miniaturization, noninvasiveness, rapid response times, excellent selectivity, and high sensitivity (Reverte et al., 2015). Utilizing the mechanism by which TTX can block action potentials in electroactive cells, such as cardiomyocytes, myocellular microelectrode array biosensors can be employed as powerful tools for studying the toxic effects of conventional cellular external field potential signals generated by TTX and for the quantitative analysis of TTX toxicity (Jahnke et al., 2013). A portable high-throughput potential biosensor based on cardiomyocytes, a 16-well microelectrode sensor, and a 32-channel recording system has been established to efficiently and quantitatively detect the toxic effects of TTX, and can rapidly detect 0.30 ng/mL TTX within 10 min (Sun et al., 2023). In addition, neuroblastoma cell lines can be used to detect marine-derived toxins and include the mouse Neuro-2a cell line, which has been used for TTX testing (Alkassar et al., 2023). In studies where Neuro-2a cells were immobilized on electrodes made of various materials and cell viability was assessed using cyclic voltammetry, it was found that carbon and carbon/polyaniline electrodes demonstrated superior results in terms of oxidation potential and current strength, enabling effective detection of TTX (Alkassar et al., 2022). Considering the mechanism of action of TTX and its analogues, an automated patch clamp (APC) system that reflects the activity of ion channels in the cell membrane can be used for TTX detection (Randall et al., 2006). Notably, an *in vitro* toxicological method involving an APC system with Neuro-2a cells has been used for the determination of TTX in puffer fish samples and the detection limits (LOD) of this method was 0.05 mg TTX equivalent/kg (Campas et al., 2024).

## 3 Detection based on immunity and immune sensors

**FIGURE 1:**
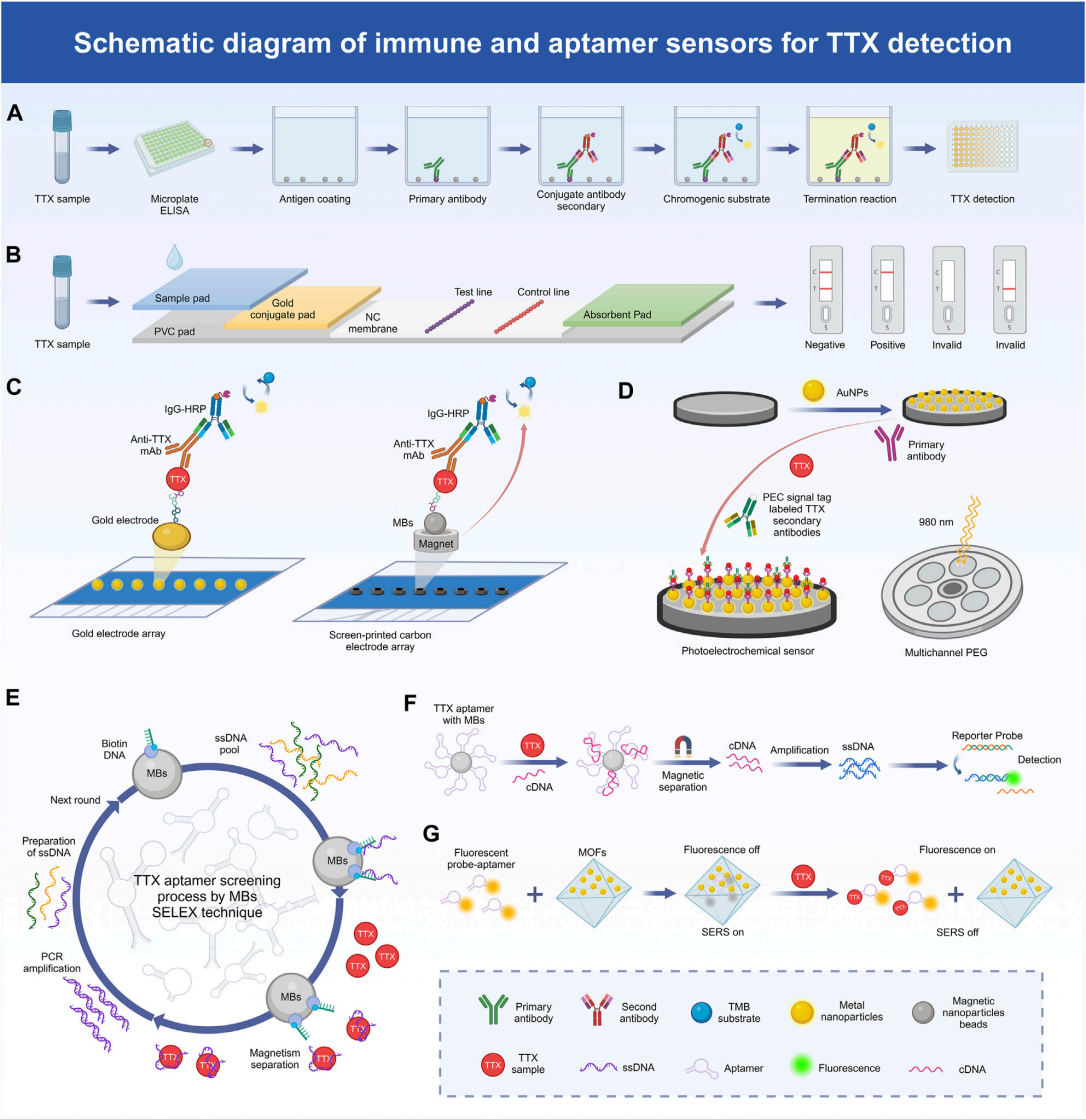
Schematic diagram of the mechanism of TTX detection method. **(A)** The process of TTX detection by ELASA technology based on immunoassay. **(B)** The process of TTX detection by colloidal gold technology based on immunoassay. **(C)** Schematic representation of the electrochemical immunosensor array platform based on gold electrode array and magnetic beads (MBs) for TTX detection. **(D)** Schematic representation of the photoelectric chemical (PEC) immunosensor based on gold nanoparticle-functionalized paper-based screen-printed electrode (PSPEG) for TTX detection. **(E)** TTX aptamer screening process by SELEX technique. **(F)** Mechanism of TTX detection method based on MBs-aptamer competition system. **(G)** Mechanism of TTX detection using a two-mode aptamer sensor based on metal-organic frameworks (MOFs).

Immunoassays and immunosensors exhibit excellent specificity and sensitivity, coupled with affordability, user-friendliness, and swift operation. In this regard, the enzyme-linked immunosorbent assay (ELISA) stands out as the primary immunoassay method (Figure 1A). Monoclonal antibodies (McAbs), renowned for their exceptional specificity and affinity, find extensive application within the ELISA (Tao et al., 2010). Additionally, immunoassays based on colloidal gold nanoparticle probes are significantly faster than the traditional ELISA and can be completed within 10 min (Zhou et al., 2010) (Figure 1B). TTX IgG McAb with a high titer has been obtained using TTX-bovine serum albumin and the TTX-keyhole limpet hemocyanin conjugate as the antibody and immunogenic antigen, respectively. A highly sensitive and reproducible ELISA method has been developed to detect TTX, the linear range of TTX was 5–500 ng/mL, and the LOD was 4.44 ng/mL. Simultaneously, researchers have developed a colloidal gold nanoparticle probe and immunoassay for the rapid detection of TTX (Ling et al., 2015). In addition, researchers have developed a modified enzyme-linked immunosorbent assay (mELISA) based on the fixation of TTX via a self-assembled dimericcarboxylate monolayer on a maleimide plate, facilitating ordered and directed antigen–antibody fixation and affinity interactions. Compared with surface plasmon resonance analysis, liquid chromatography-tandem mass spectrometry (LC-MS/MS), and the MBA, this method exhibits a good detection effect, and the LOD of TTX is 0.23 mg/kg (Reverte et al., 2015). Furthermore, a separate study introduced an mELISA by leveraging a dithiol self-assembled monolayer (SAM). This innovation shifted the SAM-based approach from the use of a microtitration plate to a gold electrode array, facilitating the transition from colorimetric immunoassays to electrochemical immunosensors. This established a targeted and reliable platform for antigen-modified sensing, suitable for analyzing natural TTX samples. The electrochemical immunosensor is capable of measuring TTX at levels as low as 0.07 mg TTX equivalent/kg (Reverte et al., 2017). Another study opted for a cysteamine-based SAM rather than a dithiol one, reducing the assay time and costs while maintaining sensitivity in detecting TTX levels in oyster and mussel samples (Reverte et al., 2018).

Surface plasmon resonance (SPR) arises within conductive films (e.g., Au) at the juncture of a medium with distinct refractive indices (e.g., sensor chip and sample solution). The wavelength shift in SPR reveals the interplay between the dissolved analyte and the biomolecule affixed to the sensor surface. SPR sensors facilitate real-time, label-free analysis devoid of hazardous solvents, risky radiolabels, or reliance on animal systems (Homola, 2008). This immunoassay method has also been transferred to commercial instruments, where it has been optimized and improved for the rapid detection of TTX (Yakes et al., 2011). One study developed an SPR optical biosensor technology for rapid screening of TTX (Campbell et al., 2013), while another study developed a direct SPR immune sensor targeting TTX to achieve direct determination of small molecular toxins TTX in seafood (Yakes et al., 2014). Additionally, a widely used point-of-care testing method is the lateral flow immunochromatographic strip (LFICS). The conventional competitive LFICS employs gold nanoparticles (AuNPs) as a signal reporter to identify small molecules, yet it tends to exhibit modest sensitivity (Ling et al., 2020). Gold nanoflowers (AuNFs) and latex microspheres (LMs) offer the benefits of a high surface area-to-volume ratio and straightforward preparation. Researchers have utilized AuNF and LM probes for McAb labeling, leading to the development of immunochromatographic test strips capable of *in situ* and rapid TTX detection. The linear ranges of TTX test strip based on AuNFs and LMs were 9.49–330.98 ng/mL and 5.40–443.19 ng/mL, and the LOD were 9.49 ng/mL and 5.40 ng/mL, respectively (Huang et al., 2023). Moreover, a LFICS combining quantum dot nanobeads and AuNFs has been developed, which also has superior signal brightness and low background interference signals when detecting TTX (Shen et al., 2017).

Electrochemical biosensors boast versatility, reliability, and swift analysis times (Figure 1C). In the development of electrochemical immunosensors, fixing the recognition element to the electrode surface is important, and magnetic beads (MBs) can be used as an alternative immobilization carrier. A magnet can simply be placed below the working electrode to fix the MB immune complex to the electrode surface without affecting the sensitivity of the method. An MB-based electrochemical immunosensing tool has been developed for TTX detection, where TTX achieves stable immobilization by forming a cysteamine SAM coupled to the MBs (Leonardo et al., 2019). A colorimetric immunoassay based on MBs in suspension was also developed for the detection of TTX in Pacific oysters, razor clams and mussels. The effective LOD for TTX in oysters and clams was 1 μg/kg, and for TTX in mussels was 3.3 μg/kg (Campas et al., 2020). Another study applied flow-based systems and antibody recognition for fluid force identification to detect TTX using a micrometer-diameter MB-labeled sandwich immunoassay form (Yakes et al., 2010). Electrochemical immunosensors can also comprise ionic liquids (ILs) and carbon nanotubes (CNTs). ILs are usually composed of organic cations and different anions, serving as effective solvents for both organic and inorganic substances (Wei and Ivaska, 2008). CNTs are extensively employed in electrochemical analysis due to their distinctive attributes, including a sizable active surface area, excellent mechanical durability, and high electronic conductivity (Agui et al., 2008). A novel carbon composite electrode, comprising the IL *n*-octylpyridinum hexafluorophosphate and single-walled CNTs, has been used as the basis of an electrochemical immunosensor. Integrated with the ELISA and antigen-bound magnetic particles, this immunosensor functions as a rapid and sensitive detector capable of directly identifying TTX within 20 min, and its detection linear range is 2–45 ng/mL, with the LOD of 5 ng/mL (Zhang et al., 2016). Photoelectrochemical (PEC) immunosensors offer high sensitivity, ease of operation, and miniaturization benefits (Figure 1D). Near-infrared-responsive photosensitive materials further enhance these advantages with their excellent biocompatibility and minimal phototoxicity, thereby playing a pivotal role in advancing PEC sensor applications (Hao et al., 2021). Notably, a new PEC immunosensor utilizing gold nanoparticle-functionalized paper-based screen-printed electrodes (PSPEG) has been developed for the real-time detection of TTX with a linear range of 0.001–100 and a LOD of 5 pg/mL (Zheng et al., 2024).

## 4 Detection based on aptamer sensors

Aptamers are biometric molecules that can be used as a substitute for antibodies and are commonly used in the determination of toxins, such as TTX. An aptamer is a single-stranded synthetic oligonucleotide that, due to its specific structural conformation, is able to bind its targeted molecule with high affinity and specificity (Iliuk et al., 2011). Aptamers offer several advantages over antibodies, including the abovementioned high affinity and specificity, repeatable chemical synthesis, stability in diverse environmental conditions, reversible denaturation, and straightforward site-directed modification (Zhang et al., 2020). The *in vitro* screening of aptamers circumvents the ethical concerns linked to animal experiments involving antibody production. Additionally, it eliminates potential issues related to immunogenicity and toxicity, thereby broadening the scope of target selection (Famulok and Mayer, 2014). The exponential enrichment ligand phylogenetic technique for the *in vitro* screening of aptamers is often referred to as SELEX (Figure 1E). A highly sensitive mixed antibody–aptamer sandwich method has been developed using capture-SELEX and next-generation sequencing technology to successfully quantify TTX in puffer fish extracts (Shkembi et al., 2021). An improved multi-SELEX technique based on magnetically reduced graphene oxide has also been created to effectively screen aptamers for TTX detection (Gu et al., 2018). Moreover, in another study, researchers repurposed existing aptamers for novel applications, utilizing molecular docking to screen for TTX candidate aptamers with superior thermal stability among DNA aptamers. Subsequently, the binding efficacy of the identified candidate aptamers was validated through microscale thermophoresis experiments. Based on the selected aptamers, two variants with good thermal stability were further designed specifically for TTX, namely, Tv-46 and AI-52 (Li et al., 2022). Furthermore, a non-label fluorescent aptamer sensor for TTX has been reported, involving a TTX aptamer as the recognition unit, berberine as the signal reporter gene, and exonuclease I, and LOD up to 11.0 p.m. was reported, showing high specificity and sensitivity (Lan et al., 2020). The isothermal amplification technique exhibits high amplification efficiency and detection sensitivity and has been widely used in the detection of biological molecules, such as aptamers. Some studies have reported the preparation of magnetic nanoparticle (MNP) aptamers via the reaction of biotin and streptavidin. A newly devised method, employing MNP aptamers alongside a triple-cycle amplification technique, presents an effective and highly sensitive approach for detecting and analyzing TTX in food samples with a LOD as low as 0.265 pg/mL (Zhang et al., 2020) (Figure 1F). Furthermore, an aptamer sensor for TTX was developed on a glassy carbon electrodes (C) that was modified with poly (4-styrenesolfonic acid)-doped polyaniline film with a LOD value of 0.199 ng/mL (Fomo et al., 2015).

Metal–organic frameworks (MOFs) represent a category of exceptionally porous crystalline materials, synthesized through the ordered self-assembly of metal-based nodes and organic linkers via coordination interactions (Kumar et al., 2015) (Figure 1G). Due to the superior chemical and physical properties of nanoparticles, the encapsulation of AuNPs in MOFs has attracted wide attention. A dual-mode aptamer biosensor has been proposed, where TTX was detected via ultra-sensitive fluorescence spectroscopy and surface-enhanced Raman spectroscopy (SERS) using an AuNP-embedded MOF nanohybrid (AuNPs@MIL-101). Employing Cy3-labeled TTX-specific aptamers as both the recognition element and signal probe enabled the effective adsorption of Cy3-aptamers onto the surface of AuNPs@MIL-101, leading to fluorescence quenching and SERS enhancement. This method has demonstrated remarkable detection sensitivity and simplicity in naturally TTX-contaminated samples, and the detection sensitivity is 6 and 8 pg/mL, respectively, which significantly improves the reliability and precision of the analysis (Liu et al., 2022). In addition, a nanoprobe with a strong and stable electrochemical/SERS double signal has been designed for dual-mode detection and analysis, where an aptamer sensor with electrically active and SERS-active Ag@Cu_2_O nanoparticles was developed to achieve accurate dual-mode TTX detection. The LOD of electrochemical signal was 31.6 pg/mL, and that of SERS signal was 38.3 pg/mL (Yao et al., 2023). Furthermore, TTX nanosensors with remarkable stability, pH independence, selectivity and LOD of 3.07 nM have been developed using zirconium-based fluorescent nanoscale MOFs combined with aptamers labeled with fluorescent dyes (Dou et al., 2023). In addition, researchers have created a rate-type fluorescent aptamer sensor for the determination of TTX using a Fe/Zr bimetallic organic skeleton (ZrFe-MOF) with high peroxidase simulation activity. ZrFe-MOF enables the specific recognition and adsorption of aptamers; the aptamer binds to TTX specifically, triggering the release of a rigid complex on the surface of ZrFe-MOF and reactivating its peroxidase simulation activity, which can be used in food safety to monitor trace TTX amounts. With this detection strategy, the LOD of TTX is 0.027 ng/mL, and the linear range is 0.05–500 ng/mL (Liu et al., 2023). Besides, researchers have introduced a novel smartphone-based portable fluorescent biosensor that utilizes a zinc-based MOF biocomposite for capturing targets and measuring fluorescence responses. An Ab-immobilized cotton swab has been employed as a tool for capturing TTX, enabling quantitative results to be obtained using a smartphone with a LOD of 0.4 ng/mL (Liu et al., 2024).

## 5 Detection based on LC-MS/MS

High-performance liquid chromatography (HPLC) and LC-MS/MS are common techniques for TTX detection (Table 1). They exhibit a low detection limit and a good linear range and allow for the simultaneous identification and quantification of toxins with good sensitivity and selectivity (Chen et al., 2011). However, the substrate of marine organism tissues is notably complex, and its background interfering pollutants can affect the accuracy of TTX detection via LC-MS/MS. Immunoaffinity chromatography (IAC) purification technology serves as an effective pretreatment method before conducting LC-MS/MS, and the imprinted antibody displays high specificity to the target analyte (Senyuva and Gilbert, 2010). Researchers have also developed an efficient method for the determination of TTX in marine organisms using samples purified by IAC combined with ultra-performance liquid chromatography-tandem mass spectrometry, or UPLC-MS/MS (Zhang et al., 2015). Most analytical methods for TTX detection are intended for use with food tissue samples, and since TTX concentrations in human biological fluid samples, such as complex water-rich matrices, are usually extremely low, effective methods for their determination are lacking (Leung et al., 2011). Hydrophilic interaction chromatography (HILIC) is a liquid chromatography (LC) technique that can be used to measure TTX concentrations in complex water-rich substrates (Gama et al., 2012). It exhibits excellent TTX retention, favorable spray conditions at the liquid chromatography-mass spectrometry (LC-MS) interface, and enhanced ionization efficiency, resulting in an improved mass spectrometric response (Nováková et al., 2014). Researchers have also devised a straightforward, versatile, and automated pulse-diffusion-focusing (PDF) strategy and developed a novel automated PDF-HILIC-MS/MS system, applicable to detecting TTX levels in plasma and urine samples (Long et al., 2020).

**TABLE 1:** LC-MS methods for TTX.

Sample	Extraction	Detection method	Column	Mobile phases	LOD and LOQ	Linear range	Average recoveries	Reference
TTX in Marine biological samples	IAC column	UPLC-MS/MS	ACQUITY UPLC BEH Amide column (50 mm × 2.1 mm I.D., 1.7 mm particle size)	Acetonitrile (A) and 5 mmol L–1 ammonium acetate in ultrapure water containing 0.1% formic acid (v/v) (B)	0.1 and 0.3 ng/g	0.3–20 ng/mL	86.5%–103.6%	Zhang et al. (2015)
TTX in plasma and urine samples	Not reported	PDF-HILIC-MS/MS system	TSK gel Amide-80 column (2.1 mm × 50 mm, i.d. 3 μm)	Acetonitrile (A) and 0.1% formic acid in water (v/v) (B)	0.0086 ng/mL and 0.029 ng/mL	0.13–12.7 ng/mL	91%–113.3%	Long et al. (2020)
TTX in mussel samples	Dispersive extraction procedure	CE-MS/MS	Bare fused-silica capillary tubing (50-μm inner diameter, 363-μm outer diameter)	5 M HCOOH in 10% MeCN/H2O (v/v)	LOD: 0.0052 mg/kg	Not reported	Not reported	Beach et al. (2018)
TTX aqueous	GO-PAN@PNE SPME fibers	UPLC-MS/MS	ACQUITY BEH HILIC column (2.1 × 100 mm, 1.7 μm)	Water with 0.1% formic acid (A) and ACN with 0.1% formic acid as mobile phase B	11.8 ng/mL and 81.3 ng/mL	100–1000 ng/mL	Not reported	Meng et al. (2022)
TTX spiked fish					32 ng/g and 150 ng/g	150–1000 ng/g		
TTX in gastropods samples	Cation exchange SPE	LC-MS/MS	XBridge + TM BEH Amide column (3.0 × 150 mm, 1.7 μm)	0.1% (v/v) of formic acid in water (A) and acetonitrile (B)	0.5 μg/kg and 1 μg/kg	0.1–100 ng/mL	82.6%–94.4%	Han et al. (2023)
TTX in plasma	Three HILIC-type SPE carriers (PSA, silica, Siphila i HILIX)	LC-MS/MS	ACQUITY UPLC BEH Amide column (50 × 2.1 mm, 1.7 μm)	Deionized water (A) and ACN (B), both containing 10 μmol/L ammonium formate and 0.01% FA	LOQ: 0.1 ng/mL	0.1–20 ng/mL	Not reported	Xin et al. (2022)
TTX in human serum	QuEChERS approach		TSK-Gel Amide-80 column (i.d. = 2.0 mm × 150 mm, 5 μm)	Water containing 0.1% (v/v) formic acid and 5 mmol/L ammonium formate (A) and acetonitrile (B)	0.67–2.61 ng/mL and 2.23–8.69 ng/mL	10–200 ng/mL	85.3%–118.2%	Zheng et al. (2023)

TTX constitutes a mixture of up to four ingredients (Yotsu-Yamashita, 2001). Quantitative nuclear magnetic resonance (qNMR) can separate the signals of all TTX tautomers and can be used to accurately quantify TTX in solution (Watanabe et al., 2016). Scholars have quantified TTX and its analogues by qNMR and evaluated the chemical equilibrium relative molar reaction of TTX via HILIC-MS/MS for its accurate quantification (Watanabe et al., 2019). Moreover, capillary electrophoresis (CE) represents another analytical separation mode akin to LC, particularly adept at separating charged polar analytes. A novel, highly acidic background electrolyte has been developed for analyzing TTX in commercial CE-MS/MS systems, thereby optimizing the detection technique leveraging mass spectrometry (Beach et al., 2018).

Solid-phase microextraction (SPME) technology has no solvent requirements, is simple to operate, and exhibits rapid detection speeds, significantly reducing sample pretreatment and detection times. Combining SPME with ultra-high performance liquid chromatography-tandem mass spectrometry (UHPLC-MS/MS) leads to the relatively quick and easy *in vivo* detection of TTX in puffer fish (Meng et al., 2022). A novel poly (lactic-co-glycolic acid) SPME nanofiber was used for *in vivo* sampling of TTX in live puffer fish, with LOD ranging from 0.52 to 2.30 ng/g (Tang et al., 2018). Furthermore, an analytical method has been developed for purification by LC-MS/MS and cation-exchange solid-phase extraction (SPE) to analyze trace and extremely high levels of TTX contamination in samples (Han et al., 2023). Another study compared three HILIC-type SPE carriers with different stationary phase functional groups to develop an LC-MS/MS method for monitoring plasma TTX concentrations (Xin et al., 2022). Further, the fast, simple, inexpensive, effective, robust, and safe (QuEChERS) method is a dispersive SPE technique that can be used for the pretreatment of biological samples (Mattarozzi et al., 2016). LC in tandem with Q-Exactive Orbitrap high-resolution mass spectrometry has been combined with a modified QuEChERS procedure for the determination of TTX in human serum samples, which can be used to analyze TTX in clinical or forensic samples (Zheng et al., 2023).

## 6 Discussion

TTX, a hydrophilic low-molecular-weight neurotoxin, selectively binds to voltage-gated sodium channel receptors on nerve cell membranes. This action inhibits the transfer of sodium ions, resulting in nerve paralysis, respiratory failure, and potentially fatal outcomes, even at low doses ranging from 0.5 to 3 mg. Such toxicity levels pose a significant risk of death in humans (Ling et al., 2015). Hence, the development of precise and highly sensitive TTX detection methods is imperative for safeguarding food integrity and protecting the wellbeing of humans. Techniques employed in TTX detection include the MBA, the ELISA, electrochemical and aptamer sensing, and LC-MS assays. These detection techniques all meet the analytical requirements of TTX determination and exhibit their own advantages and applicability, though they also have corresponding inherent limitations and challenges (Supplementary Table S1).

The MBA is time-consuming, ethically limited, and inefficient, but the development of alternative biological methods has, thus far, not completely replaced this approach (Katikou et al., 2022). Cell-based biosensors can also directly reflect changes in electrophysiological functions induced by ion-channel compounds (Jahnke et al., 2013). The development of novel high-throughput cell potential biosensors has led to a noninvasive and convenient way to detect TTX in real time (Sun et al., 2023).

The conventional ELISA method, based on immune reactions, is associated with high expenses, limited reproducibility, susceptibility to false positives, and significant susceptibility to variations in temperature and duration (Ling et al., 2015). At present, significant strides have been made in TTX detection through electrochemical methodologies. The electrochemical method presents the advantages of short analysis times, good portability, low costs, high sensitivity, and signal stability (Leonardo et al., 2019). In addition, PEC sensors based on photosensitive materials are also gradually gaining widespread use and exhibit excellent detection performance (Zheng et al., 2024).

Aptamers can be used as a cost-effective alternative to antibodies and are more stable and economical than the latter. However, the aptamer biosensing platform relies on the specific recognition of the aptamer and requires complex biomolecular immobilization or modification processes to yield specific and reliable binding reactions. Most previously reported sensors are primarily based on single-sensing modes and can be affected by different measurement environments, devices, and operations (Lu et al., 2019). In contrast, the dual-mode sensing strategy is currently an option that addresses different detection conditions and that possesses a wider range of application. Moreover, the screening of TTX-specific aptamers, combined with the use of metal nanomaterials or MBs, further improves the sensitivity of aptamer sensors (Liu et al., 2022). In addition, the SERS method exhibits fast response times, convenient operation, and high sensitivity, and the dual-mode electrochemical aptamer sensor, implementing the design of electrochemical/SERS double-signal nanoprobes, also improves the accuracy and reliability of detection (Yao et al., 2023).

The traditional HPLC method can realize the sensitive and synchronous analysis of biotoxins, and LC-MS analysis has attracted increasingly greater attention because of its multifunction and sensitive detection ability (Panda et al., 2022). However, instrumental analysis methods often necessitate specialized technology, costly equipment, and labor-intensive sample preparation processes, which hinder their widespread application. Prior to analysis, the sample purification steps and the type of chromatographic separation employed play a crucial role. Further attempts to simplify the sample pretreatment process, or to optimize it by MB or paper chromatography, in combination with LC-MS detection are underway (Reverte et al., 2023). In addition, the optimization of instrumental analysis methods for TTX determination in different complex samples is an important application direction, including, for example, the optimization of HILIC detection (Leung et al., 2011). Therefore, combining different technologies for TTX detection may be favorable for creating systems that take advantage of their strengths and complement their shortcomings. Facilitating the commercialization of biosensors and optimizing high-throughput detection are also important challenges to be addressed.

## 7 Conclusion

This review summarizes detection and quantitative analysis methods developed for TTX determination. It is worth noting that the methods present both advantages and disadvantages. How to innovatively avoid the disadvantages of these detection technologies and give full play to their advantages to develop high-sensitivity, high-stability, time-saving, convenient, and economical detection methods thus forms the research focus. Using these novel biosensing tools to analyze samples and comparing them with other technologies helps validate their application potential. The bioassay tools developed thus far exhibit high performance and can effectively detect TTX in samples; these may, therefore, be used in TTX research activities and routine monitoring in the future.
