# Supplementary material for: Recent research progress in tetrodotoxin detection and quantitative analysis methods
Source: Front Chem. 2024 Aug 14;12:1447312. doi: 10.3389/fchem.2024.1447312 (PMC11349515; doi:10.3389/fchem.2024.1447312)
Supplement: Supplementary file 1 [file Table1.DOCX]

Supplementary Material

# Supplementary Table

| Method | Advantages | Disadvantages |
| --- | --- | --- |
| Mouse bioassay (MBA) | World-recognized standard methods; Can provide toxicological information; Can detect unknown toxic TTX analogues | Low accuracy due to individual differences; Low specificity; Ethical and moral issues; High cost; Low efficiency; No toxin information available |
| Cell-based biosensors | Miniaturization; Non-invasive; Quick response; Good selectivity | A large number of samples; Longer analysis time; Sensitivity difference; No toxin information available |
| Immunoassays and immunosensors | High specificity; High sensitivity; Easy to operate; Miniaturization; Business equipment | Labor-intensive; Low efficiency; High cost; Complex sample processing and antibody preparation |
| Aptamer biosensor | Strong selectivity in vitro; High affinity; High specificity; Good reproducibility of chemical synthesis; High stability; Easy specific modification；No animal experiments are involved | Complex biomolecular immobilization or modification processes are required to obtain specific and reliable binding reactions; Difficult to apply to natural sample analysis |
| LC-MS | High sensitivity; High specificity; High accuracy; Provide accurate information on toxins | Limitations on the availability of standards; Sample pretreatment is required; Time-consuming; High cost; Complex operation; Sophisticated instruments and equipment are required |

**Supplementary Table 1. Common methods for TTX detection**
